# Evidence-based selection of orthodontic miniscrews, increasing their success rate in the mandibular buccal shelf. A randomized, prospective clinical trial

**DOI:** 10.1186/s12903-022-02460-3

**Published:** 2022-09-20

**Authors:** Michał Sarul, Joanna Lis, Hyo-Sang Park, Kornelia Rumin

**Affiliations:** 1 Department of Dentofacial Orthopedics and Orthodontics, Wroclaw Medical University, ul. Krakowska 26, 50-425 Wroclaw, Poland; 2 Department of Orthodontics, School of Dentistry, Kyungpook National University, Daegu, 41940 Korea

**Keywords:** Orthodontics, Orthodontic anchorage procedures, Mandible

## Abstract

**Background:**

Skeletal anchorage has made it possible to perform complex orthodontic tooth movements that are difficult or even impossible to achieve with conventional orthodontic treatment. Mandibular buccal shelf miniscrews, used for distalization, play a particularly important role in treatment of Class III malocclusion. Unfortunately, stability of the miniscrews placed in the mandible is still considered at higher risk of failure compared to other intraoral locations. The aim of our study was to determine the influence of the miniscrew size on their long-term stability, occurrence of oral mucosa inflammation and pain lasting over 48 h after implantation.

**Methods:**

184 Absoanchor^®^ miniscrews (Dentos, South Korea) in two sizes: SH2018-10 (length 10 mm, ø 1.8–2.0 mm) and SH1514-08 (length 8 mm, ø 1.4–1.5 mm) were inserted in the mandibular buccal shelf in 92 Caucasians aged 20–50 years, diagnosed with Class III malocclusion that required en-masse distalization of the mandibular dentition. Data was statistically analyzed with the level of significance set at p = .05.

**Results:**

91.3% of the SH2018-10 and 75% of the SH1514-08 miniscrews were stable, and this difference was statistically significant (p < .05). Inflammation of the oral mucosa was noticed around both types of miniscrews and affected 50% of the SH2018-10 and 26.09% of the SH1514-08 group (p < .05). Pain lasting longer than 48 h after implantation was related to 60.87% and 20.65% of the SH2018-10 and the SH1514-08 miniscrews (p < .05), respectively. Inflammation associated with larger SH2018-10 miniscrews did not affect their stability (p > .05), contrary to the SH1514-08 ones (p < .05). When inflammation was present, the overall success rate declined to 64.29%, from 94.74% noted for TADs without inflammation. According to the log-rank test, smaller TADs failed significantly sooner than the larger ones (p = .002).

**Conclusion:**

Larger SH2018-10 miniscrews are the anchorage of choice for the mandibular buccal shelf, despite triggering inflammation and long-lasting pain significantly more often than the smaller ones. Therefore, this issue should be discussed with every patient prior to miniscrew use. *Trial registration* ID: ClinicalTrials.gov Identifier: NCT05280678 Date of Registration: 15/03/2022. Retrospectively registered.

## Background

Challenging issue of the reciprocal forces has been technically solved by temporary anchorage devices (TADs). Discovery of the osseointegration process by Brånemark et al. [1] and studies performed by Kanomi and Costa [2, 3] laid the foundations for the use of orthodontic miniscrews. Currently, TADs serve as skeletal anchorage reinforcement, their insertion and removal is easy, they are inexpensive and can be loaded immediately after placement. TADs are commonly used in orthodontic treatment, providing sufficient anchorage for demanding tooth movements, such as unilateral closure of extraction spaces, management of occlusal plane canting, intrusion of the lateral teeth or protraction/retraction of the entire dentition [4–6]. Considering treatment options in the mandible, TADs can serve as an excellent anchorage for en mass distalization in Class III patients, where tooth movement should be controlled 3-dimensionally [7]. This camouflage treatment option is an alternative to traditional orthodontic techniques, like Class III elastics resulting in unfavorable proclination of the maxillary incisors and extrusion of the molars, or lower premolars extractions that provoke excessive lingual inclination of the mandibular anterior teeth after the treatment [8].

According to systematic review and meta-analysis of Alharbi et al. [9], TADs have an acceptably low failure rate 13.5%, (95% CI 11.5–15.9). However, they are more likely to fail in the mandible: 16.5% (95% CI 11.6–22.7) than in the maxilla: 11.0% (95% CI 8.8–13.7) [9]. Papageorgiou et al. [10], Park et al. [11] and Antoszewska et al. [12] also agreed that location in mandible increases the risk of their rejection. The cortical bone in the mandible is thicker and denser than in the maxilla; regardless of this, poorer results might be caused by bone overheating during drilling and TADs irritation from chewing [11]. In contrast, Chang et al. [13] obtained superior stability in the mandible, however using miniscrews with large diameter and length (2 × 12 mm). It is in accordance with other studies, where larger miniscrew size improved stability gained via mechanical interdigitation with the thicker bone [5, 13, 14]. Sarul et al. [15] demonstrated significantly higher survival rate of 8-mm long miniscrews inserted in the mandibular buccal area for retraction purposes, comparing to the 6-mm ones. Additionally, according to a finite element study of Liu et al. [16] a wider screw diameter provided superior mechanical advantages. Both screw displacement and bone stress decreased, as the diameter of the miniscrew (1.2, 1.5 and 2 mm) and cortex thickness increased. Lu et al. [17] also reported that the effect of force on stress around the implant was related to the miniscrew diameter, but not to its length.

Regardless of superior stability reported by some authors, the larger TADs indisputably break the integrity of bone in a greater area than the small ones. Therefore, the aim of our study was:

1. To determine the influence of the miniscrew size on their long-term stability in the mandibular buccal shelf location.
2. To determine whether different miniscrew size contributes to the occurrence of oral mucosa inflammation, possibly jeopardizing TADs stability, and pain lasting over 48 h after implantation, minimizing patients’ comfort.

## Materials and methods

**Fig. 1:**
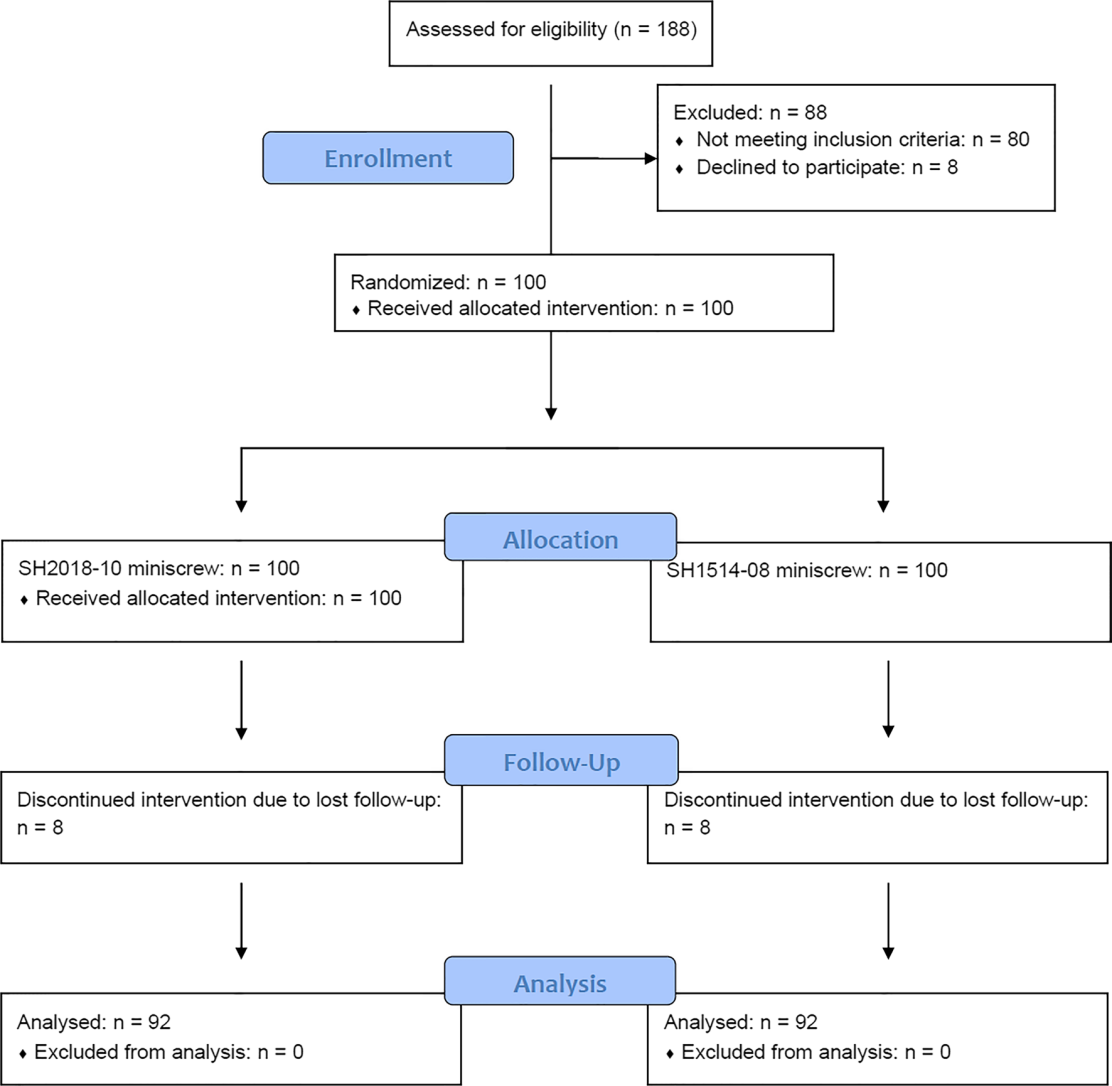
CONSORT participant flow diagram

This prospective, randomized clinical trial received approval of the Bioethics Committee of the Wroclaw Medical University (approval no. 293/2007) and was retrospectively registered at ClinicalTrials.gov (identifier: NCT05280678, date of registration: 15/03/2022). The study group consisted of 92 generally healthy Caucasian patients [49 women, 43 men, mean age 31.8 (+-7.7)], who required an absolute anchorage for en-masse distalization in the mandible. As for inclusion criteria, they comprised patients with mild skeletal Class III, with either hypodivergent or normal angle between the maxillary and mandibular planes, with excellent oral hygiene and favorable anatomical conditions (e.g. absence of a strong frenulum, potentially irritating the screw head during chewing and/or facial movement or forcing screw-head position requiring alteration of force vector directions). The trial was conducted in accordance with CONSORT guidelines (Fig. 1).

If mandibular third molars were present, their extraction was performed before treatment. Then all patients were treated with the (.022”, Roth prescription, GC Orthodontics Europe GmbH®, Breckerfeld, Germany) full fixed appliance system. Both arches were leveled with continuous wires, starting with 0.016-inch nickel-titanium, and worked up to 0.019 × 0.025-inch stainless steel over the course of several months. After that, the patients were ready for TADs placement.

**Fig. 2:**
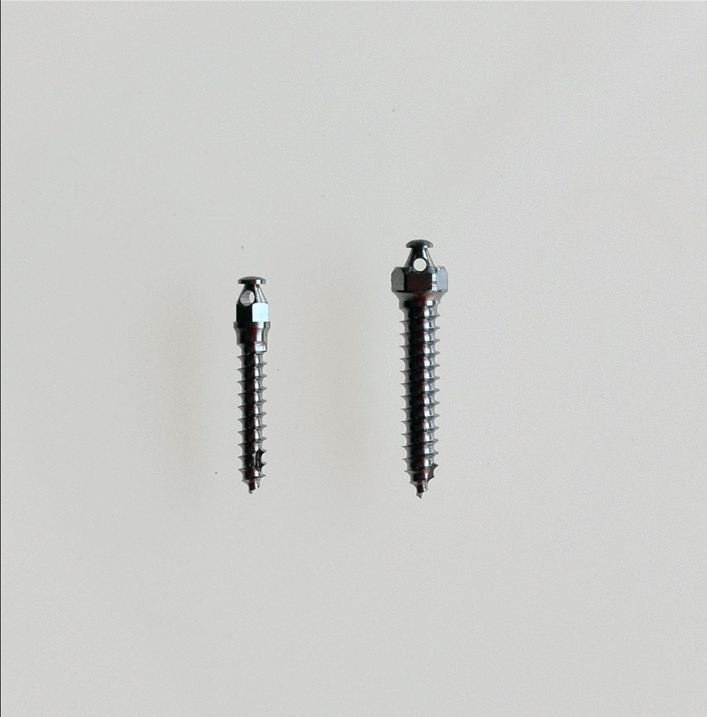
Mini-implants used in the study (from left to right): SH 1514-08 and SH 2018-10

184 miniscrews (Absoanchor^®^, Dentos, South Korea) made of titanium alloy (Ti-6Al-4 V) in two sizes have been analyzed in this study (Fig. 2): SH1514-08 (diameter of 1.5 mm at the neck, 1.4 mm at the apex and 8 mm in length) and SH2018-10 (diameter of 2.0 mm at the neck, 1.8 mm at the apex and 10 mm in length). Thus, the intraosseous parts were either 8 or 10 mm long, with a button-like head with a small hole. Biocompatible Ti-6Al-4 V TADs were used, which, while releasing very minor, clinically insignificant amounts of aluminum and vanadium, have greater mechanical strength compared to pure titanium and are best suited to a small diameter, reducing the risk of fracture during insertion and removal [18, 19].

**Fig. 3:**
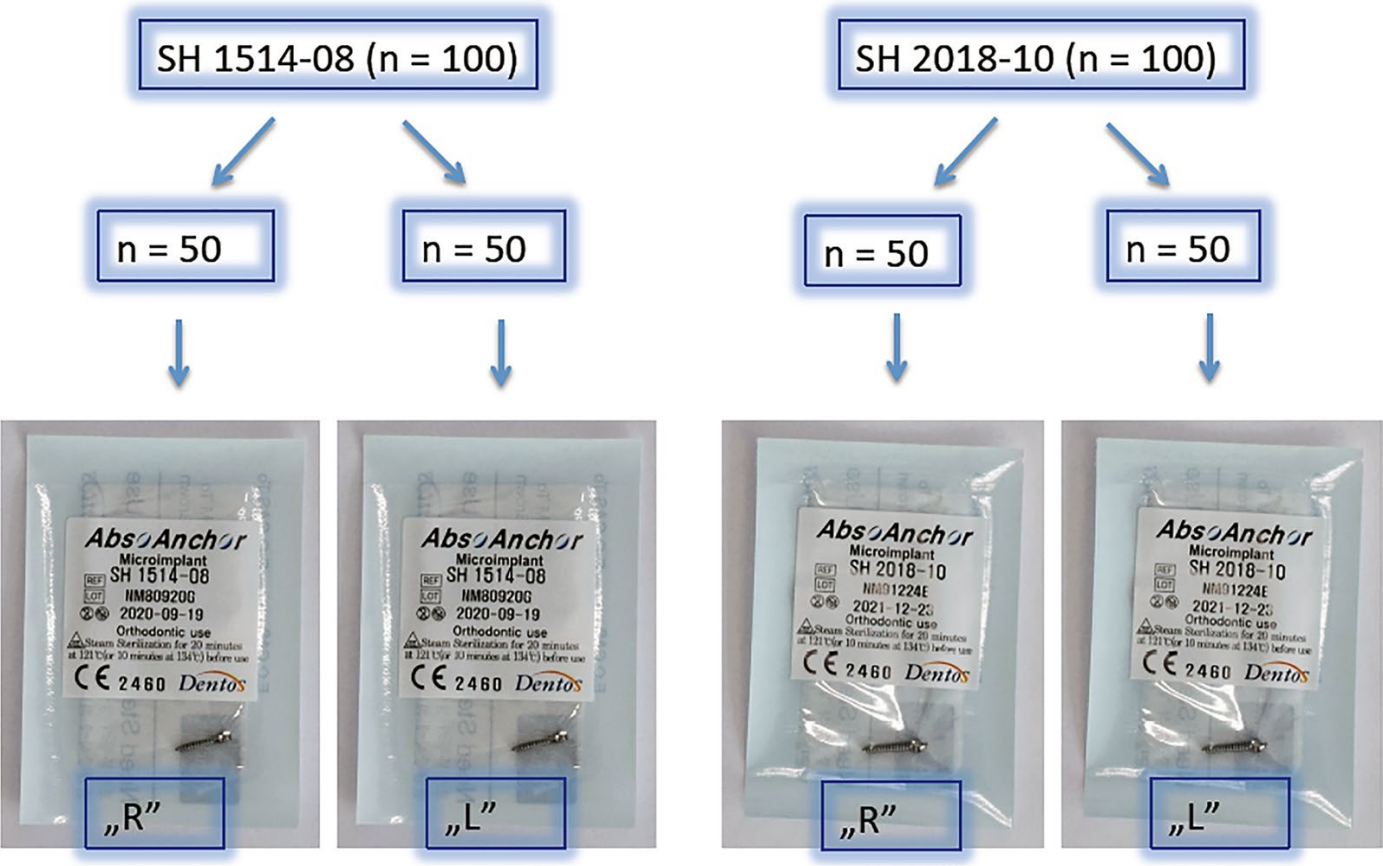
TADs allocation securing randomization. captions: R–right side, L–left side

We designed our project as a split-mouth study, therefore each patient received both SH2018-10 and SH1514-08 miniscrews. To do so, our nurse divided both TADs types into two halves (Fig. 3) and assigned symbols appropriate for blinding the intervention. Thus, two combinations of TADs sets arised: (1) SH1514-08R (right) and SH2018-10 L (left) or (2) SH1514-08 L (left) and SH2018-10R (right), which were placed separately in opaque packages marked consecutively from “1” to “100” and stored on the tray with dividers. One hundred cards, labeled accordingly, were placed in an envelope, from which the nurse blindly pulled the card just before the miniscrew insertion, assigning the set number to every patient. Therefore both placement side and screw size were random for the clinician.

**Fig. 4:**
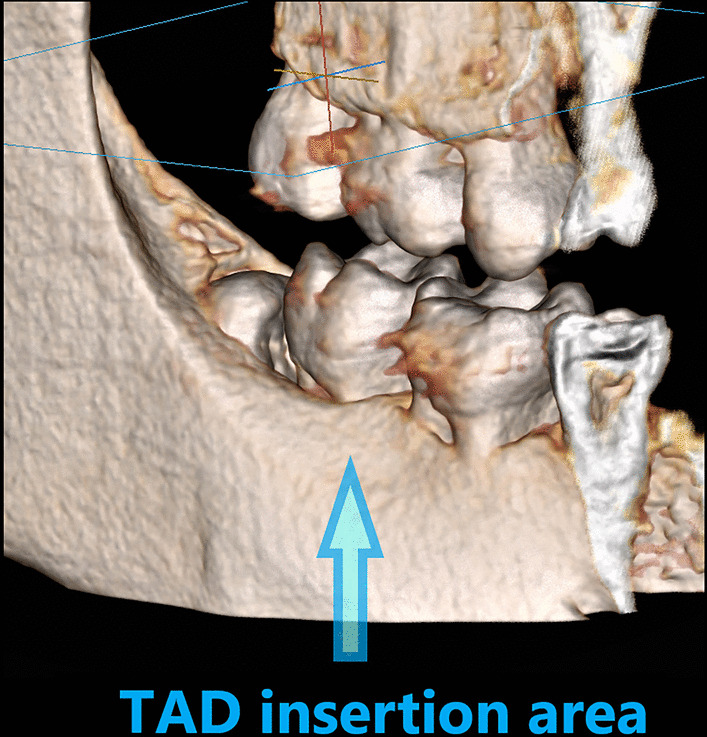
TAD insertion area

**Fig. 5:**
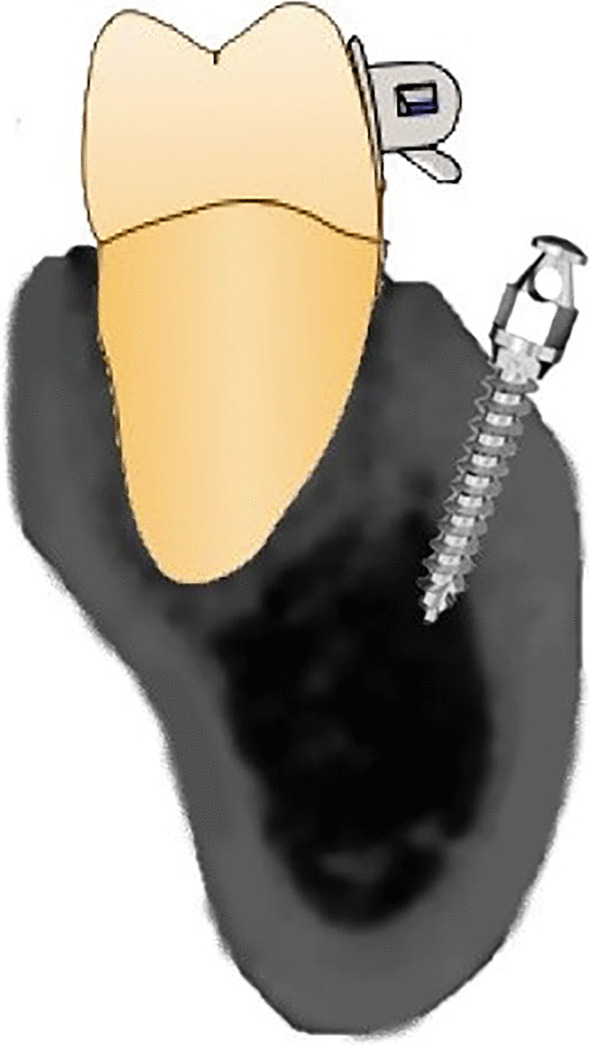
Frontal cross-section of mandibular buccal shelf illustrating axial inclination of the TAD (extra-alveolar approach)

One orthodontist (M.S.) inserted the screws near the muco-gingival junction, following one surgical protocol. Under local anesthesia, the doctor performed a vertical stab incision (3–4 mm) and made a hole using a pilot drill with a working speed of 500–1000 rpm under massive saline irrigation. Miniscrews were always placed in the mandibular buccal shelf, lateral to the first and second molar interproximal area (Fig. 4). It allowed achieving miniscrew angulation parallel to the long axis of adjacent molars (Fig. 5), as well as reducing the risk of root-contact or interference with the tooth movements. The screw head was adjusted at least 2–3 mm above the mucosa. No analgesics or antibiotics were prescribed after miniscrew placement. The patients were instructed to: maintain flawless oral hygiene, to use 0.2% chlorhexidine gel twice a day (Elugel) (Pierre Fabre Medicament Polska Ltd, Warsaw, Poland) around TADs’ head for the first 2 weeks after operation, and to avoid any hitting against miniscrews.

Miniscrews were loaded with orthodontic force (NiTi closed coil springs) of approximately 200 g two weeks after the surgery. On the same visit patients were surveyed upon pain incidence lasting longer than 48 h. Miniscrew stability and soft tissue condition were then closely examined at each appointment. TADs were considered long-term stable if they served as an anchorage until completion of distalization of the mandibular teeth. During the follow-up visits (every 4–6 weeks), miniscrew stability and condition of the surrounding soft tissue were evaluated. Hypertrophy of the gingiva and/or redness and/or tendency to bleed was noted as the inflammation presence.

Statistical analysis was performed with Statistica 12, with McNemar’s test, the chi-square test (with Yates amendment if needed) and the phi coefficient to analyze the effect size. The level of significance was set at p = .05. Cumulative survival of the SH2018-10 and SH1514-08 miniscrews over time was determined with the Kaplan–Meier analysis. Comparison of cumulative survival between two TADs sizes was performed using the log-rank test.

## Results

Success rates of the TADs were: 91.3% for SH2018-10 and 75% for SH1514-08 screws. Results of the McNemar’s test showed a statistically significant (p < .05) correlation between the miniscrew size and its success rate with a higher probability of survival for the larger SH2018-10 screws. No statistical difference was found between right and left sides (Table 1).

**Table 1:** Statistical analysis results: the overall TADs stability and the TADs stability in relation to their side of placement

TAD	Side	Success rate % (n)	The Pearson’s Chi-square test result	p value	Contingency (C) coefficient	Phi coefficient
SH2018-10		91.3 (84)	8.728653	p = 0,0031	0.2128141	−0.217803
SH1514-08		75.0 (69)				
SH2018-10	Right	91.11 (41)	0.0041427	p = .94,868	0.0067103	0.006710
	Left	91.49 (43)				
SH1514-08	Right	76.60 (36)	0.1304965	p = .71,792	0.0376355	0.0376622
	Left	73.33 (33)				

Inflammation of the oral mucosa was noticed around both types of the miniscrews, affecting 50% of the SH2018-10 and 26.09% of the SH1514-08 miniscrews; this difference was statistically significant (p < .05) (Table 2).

**Table 2:** Statistical analysis results: comparison of the TADs in terms of inducing inflammation of oral mucosa

TAD	Inflammation incidence % (n)	The Pearson’s Chi-square test results	p value	Contingency coefficient (C)	Phi coefficient
SH2018-10	50.0 (46)	11.15990	0.00084	0.2391304	0.246276
SH1514-08	26.09 (24)				

Pain lasting longer than 48 h after implantation occurred three times more frequently after insertion of the larger TADs. This pain accompanied 60.87% and 20.65% of the SH2018-10 and the SH1514-08 implanted miniscrews, respectively. The difference was statistically significant (p ≤ .001) (Table 3).

**Table 3:** Statistical analysis results: comparison of the TADs in terms of inducing pain lasting longer than 48 h

TAD	pain incidence % (n)	The Pearson’s Chi-square test results	p value	Contingency coefficient (C)	Phi coefficient
SH2018-10	60.87 (56)	30.81297	0.00000	0.3787359	−0.409221
SH1514-08	20.65 (19)				

Results of the Pearson’s Chi-square test showed that a statistically significant risk of failure due to inflammation was only related to SH1514-08 screws (p ≤ .001). Statistically, inflammation did not cause a higher failure rate of the SH2018-10 screws (Table 4). When inflammation was present, the overall success rate declined to 64.29%, from 94.74% noted for TADs without inflammation.

**Table 4:** Results of statistical significance of TADs failure risk posed by the inflammation of oral mucosa

TAD	Stability	Inflammation incident % (n)		The Pearson’s Chi-square test result	p value
		Present	Absent		
SH2018-10	Failure	10.87 (5)	6.52 (3)	0.5476190 0.1369048 *	0.45929 0.71138 *
	Success	89.13 (41)	93.48 (43)		
SH1514-08	Failure	83.33 (20)	4.41 (3)	58.92810	0.00000
	Success	16.67 (4)	95.59 (65)		
SH2018-10 and SH1514-08	Failure	35.71 (25)	5.26 (6)	28.70611	0.00000
	Success	64.29 (45)	94.74 (108)		

*With Yates amendment

**Fig. 6:**
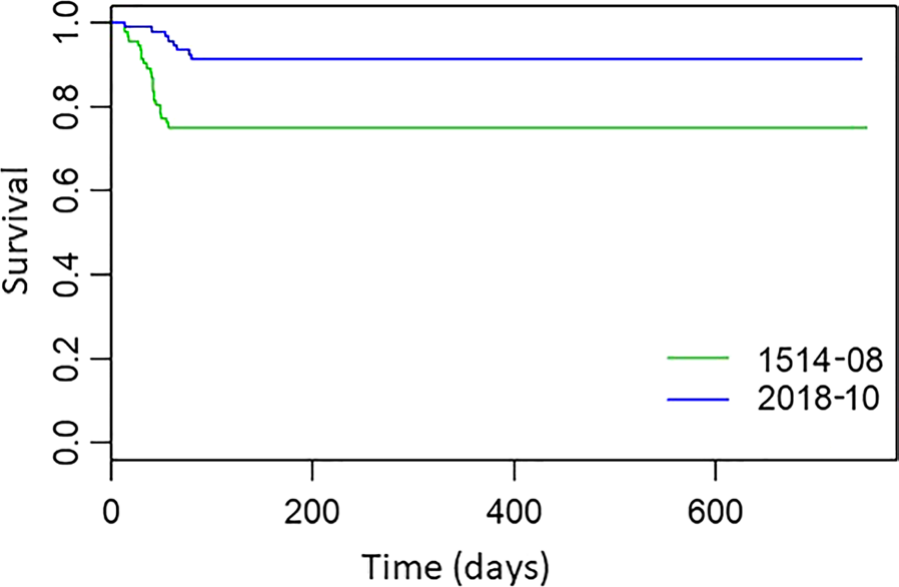
Survival distribution of the mini-implants with respect to their size: SH1514-08 and SH2018-10.

The results of the Kaplan-Meier survival analysis over time between SH2018-10 and SH1514-08 miniscrews are presented in Fig. 6. According to the log-rank test, smaller TADs failed significantly sooner than the larger ones (p = .002).

## Discussion

Unfortunately, the use of TADs still carries a certain risk of failure distinctly varying in individuals [6, 20, 21]. Patient’s characteristics and local bone quality are often listed as the critical factors [6, 21]. Therefore, TADs stability in the mandible or in the maxilla may differ substantially. Kuroda et al. [20] and Tseng et al. [21] reported that skeletal anchorage remains stable more often in the maxilla than in the mandible. Park et al. [11] and Chen et al. [22] came to similar conclusions placing the TADs distally in the alveolar part of the mandible. Meta-analysis carried out by Hong et al. [14] also confirmed that stability of the TADs placed in the mandible is 2.23 times lower than in the maxilla, which is a statistically significant difference. However, the multiple data demonstrating that achieving stable TAD position in the posterior part of the mandible vary substantially: from 66.7 to 92.8% of cases [12,13,15,20,21], fully justifying the aim of our study.

Miyawaki et al. [23] tested mini-implants of different sizes in the mandible. The authors showed that the TADs stability improved as the diameter of the screws got larger. Chang et al. [13], quoted in the introduction of this paper, also achieved very high success rate, namely: 92.8% stability of 1680 stainless steel (2 mm ⨯ 12 mm) miniscrews inserted in the mandibular buccal shelf, parallel and distal to the lower first and second molar roots. On the contrary, in the study by Manni et al. [24] the smaller miniscrews (1.3 mm ⨯ 11 mm) showed significantly higher success rate than the larger ones (1.5 mm ⨯ 9 mm and 1.5 mm ⨯ 11 mm). However, the previous mini-implants were placed in both jaws, mainly in the anterior area (intra-alveolar approach), where the cortical bone is thin and the distance between the adjacent teeth is relatively short. Such anatomy somehow forces the use of small diameter screws and immediately favors rejection of the larger ones.

Despite smaller mini-implants are easier to insert between the roots, minor reduction in their size declines the torsional strength significantly and can increase the risk of implant fracture. Therefore, it is advisable to avoid miniscrews smaller than 1.2 or 1.3 mm in diameter when placing into the thick mandibular cortical bone, where miniscrew fracture is more likely to occur [6, 17, 20–25]. A meta-analysis performed by Hong et al. [14] also showed that increasing the TAD diameter above 1.4 mm gives a 1.61 times greater chance of stability. It is in accordance with our study, where 91.3% of the SH2018-10 screws were stable compared to 75% of the SH1514-08 screws. It should be emphasized that despite a larger TADs diameter we did not violate biomechanics thanks to appropriate miniscrew location (extra-alveolar approach), that enabled bodily tooth-movement of the mandibular dentition.

Oral mucosa inflammation can result in progressive damage of the cortical bone surrounding the implant’s neck, which endangers its stability [26]. Studies demonstrated that incidence of inflammation statistically contributes to mini-implant loss [27], which is partially in accordance with our study, since it concerned only smaller miniscrews. SH1514-08 screws were nearly 8-times more likely to fail due to inflammation compared with the SH2018-10 ones. Failure of the larger SH2018-10 screws due to inflammation was of no statistical significance (Table 4), nonetheless they caused inflammation in half the cases. Despite the inflammation, the larger TADs were less likely to fail, probably due to their higher bone-miniscrew contact ratio and better mechanical interlocking compared to SH1541-08 ones. In our study, we utilized the optimal position for the TADs according to Chang [13]: lateral to the first and second molar interproximal area, approximately 5 mm from the alveolar crest, and insertion at an angulation of about 30° to the bone surface. According to CBCT measurements of mandibular buccal shelf in Class III patients at a 30° angle for sites 3–7 mm from the alveolar crest, angulating the TAD in comparison to perpendicular approach consistently increased bone contact from 0.56 to 1.23 mm, which was a ~ 25–30% increase at all sites. This was an important consideration, since even a 0.5 mm difference in cortical bone thickness (bone-miniscrew contact ratio) can affect the success rate. The median for inclined cortical bone thickness at the recommended sites ranged from 3.54 to 4.05 mm, which was more than sufficient for primary stability, particularly valuable for the entire arch distalization, which itself requires a stable anchorage [13].

It is known from the literature that irritation around TADs placed in the posterior part of the mandible can be triggered by chewing [11], therefore the attached gingiva is recommended for TADs location in order to avoid interference with the functional movements of the soft tissues and—subsequently—their inflammation [28]. However, larger TADs cannot be inserted into the inter-radicular spaces. For this reason the other option proposed by Chang et al. [13], namely placing miniscrews in elevated position with the screw head at least 5 mm above the soft tissue level, is promising in terms of preventing peri-screw inflammation. Nevertheless, it does not exempt from providing instruction of oral hygiene and monitoring the condition of soft tissues at each appointment in order to reduce the risk of inflammation. Since the stability of the smaller miniscrews was significantly impaired by inflammation, even minor precautions should be taken into consideration. In our study, all patients were instructed to use chlorhexidine gel for two weeks after TADs insertion due to its antibacterial properties, that minimize risk of tissue inflammation, and its ability to slow down epithelialization, reducing the likelihood of soft-tissue overgrowth [29]. Regarding details, better hygiene is often achieved on the left side in right-handed patients, who constitute most of the population [30]. Park et al. [11] stated that TADs placed on the left side exhibited higher success rates than those placed on the right side. However, in our study no statistical difference was found between both sides.

Nevertheless, even when perfect hygiene is maintained and implantation properly performed, small fraction of patients may have a genetic predisposition to TADs failure, especially when they fail bilaterally [13]. Andrucioli et al. [31] evaluated the gene expression of proinflammatory cytokines and osteoclastogenesis mediators in peri-miniscrew gingival tissue samples using real-time polymerase chain reaction, to verify if gingival inflammation and bone resorption could be associated with implant failure. They concluded that the higher IL-6 expression could be associated with miniscrew failure, since a prolonged excessive release of IL-6 was translated into persistent oral inflammation and tissue destruction via proteases, osteoclasts and methylation changes. There are several polymorphisms in the promoter region of IL-6, and among them the IL-6 174 GG genotype plays as a risk factor of chronic periodontitis in Brazilian and Caucasian population [32]. Patients genetically predisposed to periodontitis may have the higher risk of miniscrew failure. Excessive and sustained production of IL-6 is also associated with a variety of inflammatory diseases like rheumatoid arthritis, Castleman disease, systemic-onset juvenile idiopathic arthritis or cytokine releasing syndrome [33]. Potential genetic complications constitute crucial considerations for informed consent since in case of TADs failure alternate treatment methods may be desirable: extractions, headgear or orthognathic surgery.

Pain lasting longer than 48 h is an unfavorable phenomenon, which was also observed by Miyawaki et al. [23] and Kuroda et al. [20]. In the latter study, Kuroda et al. [20] used two types of miniscrews and one type of miniplate. They observed that over 60% of the patients with the larger screws reported pain in the third day after the implantation. This result is similar to ours: smaller miniscrews were significantly better tolerated than the larger ones. Kuroda et al. [20] believed that the muco-periosteal flap reflection was the pain-causing factor. Our results seem to decline such concept: the patients experienced pain regardless our flap-less protocol. Furthermore, despite the reports that the level of prolonged pain after TAD insertion is comparable with the one associated with tooth extraction [34], or even with discomfort related to the initial tooth alignment [35], the issue of postoperative pain threshold is very individual and, thus can’t be disregarded.

In our study, the Kaplan-Meier survival analysis demonstrated that the SH1514-08 miniscrews failed significantly sooner compared to the SH2018-10 ones (p = .002). In the study by Wiechmann et al. [36], majority of the miniscrews with two different diameters (1.1 and 1.6 mm), placed buccally in the mandible failed within 50 days after placement, which is in accordance with the findings of Garfinkle et al. [37], who used 1.6 × 6 mm miniscrews. On the other hand, Chang et al. [13], who evaluated larger 2 × 12 mm miniscrews, reported average failure time of 3.3 months, which was similar to 3.4 months reported by Park et al. [11], evaluating smaller mini-implants with a diameter of 1.2 mm, 5–10 mm long. In our study majority of the miniscrew failures occurred within one to three months from their insertion, after which time success levels remained constant throughout the rest of the treatment. The survival curve was more steep and erratic for smaller TADs than for the larger ones. This observation lends credence to the theories suggesting that by increasing the TAD size one can achieve improved stability.

## Conclusion

We provided the evidence that larger SH2018-10 miniscrews are the anchorage of choice as for the placement in the mandibular buccal shelf, out of the alveolar part, lateral to the first and second molar interproximal area, as they demonstrated a significantly higher success rate (91.3%) compared to the smaller SH1514-08 ones (75%).

Inflammation associated with the miniscrews did not affect stability of the larger TADs, contrary to the smaller ones, which were more prone to failure. For this reason, every precaution should be taken to prevent peri-miniscrew inflammation by emphasizing proper oral hygiene regimen and, avoiding irritation of TADs.

Since larger TADs trigger inflammation and long-lasting pain considerably more often than SH1514-08 miniscrews, this issue should be discussed with every patient prior to the TADs use.

## Data Availability

The data that support the findings of our study are available from the corresponding author upon reasonable request.
